# Shortfalls of free autologous internal limiting membrane transplantation for highly myopic refractory macular holes in a long term follow-up

**DOI:** 10.1007/s00417-024-06533-7

**Published:** 2024-06-04

**Authors:** Matteo Mario Carlà, Carlos Mateo

**Affiliations:** 1 Ophthalmology Department, “Fondazione Policlinico Universitario A. Gemelli, IRCCS”, Largo A. Gemelli, 8, Rome, 00168 Italy; 2 Ophthalmology Department, Catholic University “Sacro Cuore”, Rome, Italy; 3 Instituto de Microcirugía Ocular (IMO), Barcelona, Spain

**Keywords:** Highly myopic macular hole, Myopic traction maculopathy, Internal limiting membrane, Autologous ILM transplantation, Autologous ILM flap, Excessive gliosis

## Abstract

**Background:**

The aim of this study is to evaluate long-term anatomical and functional outcomes of autologous internal limiting membrane (ILM) transplantation in refractory highly myopic macular holes (HMMHs).

**Methods:**

Retrospective interventional analysis of 13 eyes with refractory HMMH undergoing autologous ILM transplantation with gas tamponade. Best-corrected visual acuity (BCVA, Snellen), optical coherence tomography and fundus photography were scheduled at baseline and every follow-up visit (1, 3, 6, 12, 18, 24 months and the most recent). Preoperatively, we collected minimum linear diameter (MLD) and basal diameter (BD). Post-operatively, rates of external limiting membrane (ELM)/ellipsoid zone (EZ) restoration, excessive gliosis and subfoveal retinal pigmented epithelium (RPE) atrophy were evaluated.

**Results:**

Average AXL was 31.45 ± 2.07 mm and mean follow-up was 47.2 ± 31.4 months. Anatomical success was reached in 7/13 eyes (54%), while 2 cases showed persisting HMMH, 2 cases had early recurrence and 2 cases late recurrence. BCVA went from 0.19 ± 0.18 to 0.22 ± 0.20 at final follow-up (*p* = 0.64), improving in 5/13 eyes (38%). One eye showed continuous ELM and EZ lines, while another eye showed an irregular ELM but no EZ. Post-operatively, 5 eyes (71%) developed progressive atrophy of the subfoveal RPE, while excessive gliosis was reported in 3 eyes (43%). Furthermore, one patient developed post-operative chronic macular edema-like changes in the perifoveal area.

**Conclusion:**

Autologous ILM transplantation showed controversial anatomical outcomes and and poor visual results in refractory HMMH. Moreover, progressive subfoveal patchy atrophy and excessive gliosis are possible post-operative complications.

**Supplementary Information:**

The online version contains supplementary material available at 10.1007/s00417-024-06533-7.

## Key messages

***What is known:***

• Various surgical techniques have been proposed to treat refractory highly myopic macular holes (HMMHs), including autologous internal limiting membrane (ILM) flap transplantation, autologous retinal transplantation and lens capsule transplantation.

• Long-term analysis of the outcomes of autologous ILM transplantation still lacks in HMMH.

***What is new:***

• Primary anatomical success was reached in 54% of cases and only 38% of eyes showed improvements in visual outcomes.

• Progressive subfoveal patchy atrophy and excessive gliosis were two of the most frequent adverse effects, negatively influencing visual outcomes.

**Supplementary Information:**

The online version contains supplementary material available at 10.1007/s00417-024-06533-7.

## Introduction

In high-income countries, degenerative myopia is a leading cause of legal blindness [1]. The spectrum of the myopic traction maculopathy (MTM), which includes inner and outer retinal schisis, foveal detachment, and lamellar or full-thickness highly myopic macular holes (HMMHs), is determined by the excessive elongation of the globe. In fact, in the posterior staphyloma, the anteroposterior traction due to the strong adhesion of the vitreous cortex combines with the tangential traction brought on by a tightened internal limiting membrane (ILM) [2–4]. Specifically, the formation of HMMHs, developing in around 8% of cases of MTM, relies on the breakdown of outer retinal layers and the creation of foveal cysts, caused by the coexistence on tangential and anteroposterior forces [5, 6]. Moreover, the persisting vitreous cortex traction, typical of highly myopic eyes, may lead to the development of macular hole retinal detachment (MHRD) [7–9]. 

The introduction of ILM inverted flap more than 10 years ago has significantly improved the closure rate of HMMHs, reaching around 90% even in cases complicated by MHRD [10, 11]. Nevertheless, more than 5% of myopic patients develop refractory or recurrent HMMH after first surgery [12]. In these cases, various surgical techniques have been proposed, including autologous transplantation of a free ILM flap [13], autologous neurosensory retinal flap transplantation [14], lens capsule transplantation [15] and, more recently, subretinal human amniotic membrane (hAM) patch [16, 17]. 

Autologous ILM was successfully positioned over regions of choroidal atrophy and over posterior retinal tears originating from the staphyloma in extremely myopic RDs [18], and a similar technique was reported for perifoveal macular holes (MHs) [19]. However, as far as we know, an analysis of surgical outcomes of autologous ILM flap transplantation in the setting of refractory HMMHs still lacks. Consequently, the aim of this research was to assess long-term functional and anatomical outcomes of pars plana vitrectomy (PPV) and free ILM transplantation in highly myopic patients with refractory MHs who already underwent ILM peel. Moreover, we focused on side effects and drawbacks of this technique.

## Materials and methods

We conducted a retrospective mono-centric interventional analysis of patients undergoing PPV and autologous ILM transplantation to solve refractory HMMHs at the Instituto de Microcirugia Ocular, Barcelona, Spain, between January 2014 and June 2023. This research adhered the tenets of the Declaration of Helsinki and was approved by the Ethical Committee of the Instituto de Microcirugia Ocular, Barcelona. All patients signed an informed consent for data publication.

High myopia was defined as an axial length (AXL) > 26.50 mm or refractive error > 6 diopters (D). The following inclusion criteria were considered: presence of refractory HMMH with or without MHRD, surgical treatment with PPV and autologous ILM flap transplantation, a minimum 12-month follow-up time. The exclusion criteria were as follows: amblyopia, severe glaucoma, active uveitis, optic neuritis, severe cataract impairing retinal imaging, and serious systemic illness impacting ocular health. Out of 325 HMMHs undergoing surgical treatment in the study period, 13 eyes fulfilled the inclusion criteria. A flowchart of the selection process is visible in Supplemental file 1.

A complete ophthalmological anamnesis was carried out at the baseline visit, followed by a comprehensive ocular examination after pupil dilation and AXL measurement (IOLMaster 500, Carl Zeiss Meditec, Germany). At each follow-up, measurements of BCVA (Snellen equivalent), intraocular pressure (IOP), spectral domain (SD)-OCT assessment using the Cirrus 5000 high-definition OCT (Carl Zeiss, Dublin, CA) and fundus photography, using either ultra-wide Optomap (Optos GmbH, Düsseldorf, Deutschland) or Topcon TRC-NW8 fundus camera (Topcon, Tokyo, Japan), were among the examinations performed. Follow-ups were scheduled at 1, 3, 6, 12, 18, 24 months after surgery and at the most recent visit. Every patient completed a minimum of 12 months follow-up.

### Surgical procedure

The same skilled vitreoretinal surgeon (C.M.) performed all surgeries using a 23-gauge system (DORC Eva Nexus, DORC, Zuidland, The Netherlands). In order to confirm earlier ILM peeling, Brilliant Blue G was initially injected within the arcades around the HMMH. Perfluorocarbon liquid (PFCL) was instilled to cover the macular area. The ILM transplant was harvested using a pinch and grasp technique from a region inside the vascular arcades, beginning from the edge of the previously removed ILM and moving in a circular fashion for about one disk diameter. Then, the ILM plug was tucked inside the HMMH under the PFCL tamponade and gently massaged to ensure correct positioning with a 23-gauge soft-tipped flute needle. If the first piece of the plug was not large enough to fill in the HMMH, a second piece of the ILM graft was harvested using a similar technique. At the end of the surgery, PFCL was removed and a fluid gas exchange using 20% sulfur hexafluoride (SF6) was carried out. The patients were asked to keep a face-down position overnight and a prone position for the first 3 post-operative days.

### Scan protocol

For OCT analysis, the following scan procedures were performed for each patient: 6 × 6 mm Macular Cube, HD Cross and horizontal and vertical HD Raster 5- or 21-line. In order to guarantee the quality of each picture, the OCT scans were performed at least twice. In a fovea-centered horizontal B-scan, the caliper tool was used to measured baseline minimum linear diameter (MLD) and basal diameter (BD): the former was measured at the narrowest distance between the broken margins of the neuroepithelia with a line roughly parallel to the retinal pigment epithelium, while the latter was defined as the length of the RPE with detached photoreceptors. In cases of MHRD, the calculation of BD was not possible. In the post-operative period, anatomical closure of the HMMH was described as the disappearance of the foveal defect and no bare RPE exposed to the vitreous. Reconstitution of the external limiting membrane (ELM) or ellipsoid zone (EZ) was defined as the presence of the corresponding continuous hyperreflective line in the fovea. Moreover, rates of excessive post-operative gliosis (hyperreflective lesion with a “peak-like” aspect extending from the RPE, filling the foveal defect and ending above the retinal surface) and RPE atrophy were reported.

In the post-operative period, eyes in which the HMMH was still open at 1-month follow-up were defined as persisting HMMH, eyes in which the HMMH recurred within the first 6 months were defined as “early recurrent”, while eyes with HMMH recurring after the 6-month follow-up were defined as “late recurrent”.

The main endpoint of this study was to define anatomical and visual outcomes of autologous ILM flap in refractory HMMHs. Moreover, we focused on the evolution of ILM flap during the post-operative period.

### Statistical analysis

Statistical analysis was performed using IBM SPSS software, version 27.0 (SPSS Inc, Chicago, IL). The Shapiro-Wilk test was employed to assess the normality of the sample. Multiple comparisons test with Dunnet’s correction was used for continuous variables comparison between baseline and postoperative data. Correlation analysis was performed using Spearman coefficient. *P* < 0.05 was deemed statistically significant.

## Results

Thirteen eyes of 13 patients were included in this study. Mean age at presentation was 69.0 ± 11.1 years, male/female ratio was 3/10, while 46% were right eyes. Mean number of previous HMMH surgeries was 1.2 ± 0.6 and mean subjective symptoms duration was 3.9 ± 3.5 months. Average AXL was 31.45 ± 2.07 mm. Patients were followed for 47.2 ± 31.4 months on average (range 12–120). Mean MLD at baseline was 484 ± 238 μm. Two cases (15%) initially presented with a MHRD. Patient list with demographic, clinical and OCT characteristics is visible in Table 1. None of the patients suffered intraoperative complications.

**Table 1:** Clinical and OCT characteristics of patients with refractory HMMH undergoing autologous ILM transplantation

ID	Age (Years)	Gender	Laterality	Lens status	Spherical equivalent (Diopters)	Axial length (mm)	Previous HMMH surgery	Symptoms duration (months)	Baseline BCVA (Snellen equivalent)	MLD (µm)	BD (µm)	Primary HMMH closure	HMMH reoperations	Follow-up duration (months)	Final BCVA (snellen equivalent)	Complications
01	72	F	OS	Pseudophakic	-1.5	30.20	1	1	20/67	258	811	No	1	24	20/125	Late HMMH recurrence
02	63	F	OS	Pseudophakic	-1.5	34.45	1	1	20/100	526	554	Yes	0	54	20/100	Excessive gliosis, RPE atrophy
03	51	F	OS	Pseudophakic	-2.25	31.08	2	12	20/4000	389	512	No	1	18	20/100	Late HMMH recurrence with MHRD
04	89	F	OD	Pseudophakic	-2.75	31.27	1	1	20/400	330	MHRD	Yes	0	12	20/400	Excessive gliosis
05	67	F	OS	Phakic	-30	33.24	1	8	20/400	808	946	No	0	68	20/200	Persisting HMMH
06	61	F	OD	Phakic	-11.5	28.69	3	6	20/400	739	1103	No	0	120	20/200	Persisting HMMH
07	66	F	OS	Pseudophakic	0.5	31.06	1	3	20/32	341	572	No	0	18	20/40	Early HMMH recurrence
08	68	F	OS	Pseudophakic	0	34.91	1	2	20/1000	824	1036	Yes	0	69	20/400	RPE atrophy
09	70	M	OD	Pseudophakic	-7.25	28.97	1	1	20/200	748	789	No	0	45	20/500	Early HMMH recurrence
10	71	F	OS	Pseudophakic	0	32.87	1	7	20/125	597	1135	Yes	0	12	20/40	RPE atrophy
11	74	M	OD	Pseudophakic	-1.25	29.95	1	1	20/50	364	716	Yes	0	75	20/50	RPE atrophy, ME
12	55	F	OD	Pseudophakic	-2.5	29.15	1	5	20/50	129	632	Yes	0	51	20/100	mCNV development
13	89	M	OD	Pseudophakic	-4	32.96	1	2	20/125	400	MHRD	Yes	0	48	20/4000	Excessive gliosis, RPE atrophy

*HMMH* highly myopic macular hole, *ILM* internal limiting membrane, *BCVA* best-corrected visual acuity, *OCT* optical coherence tomography, *MLD* minimum linear diameter, *BD* basal diameter, *MHRD* macular hole retinal detachment, *RPE* retinal pigmented epithelium, *ME* macular edema

**Fig. 1:**
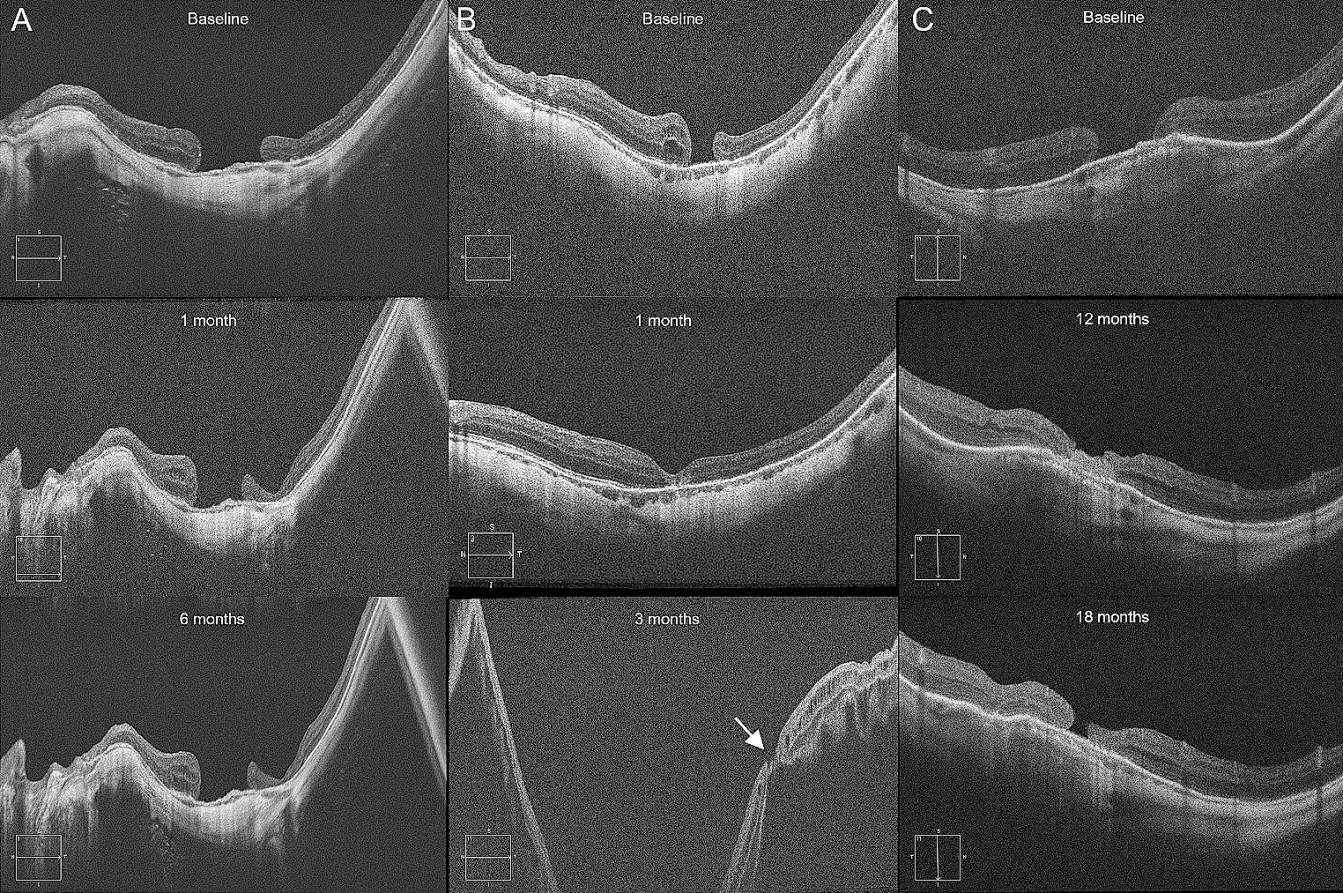
B-scans of cases undergoing autologous internal limiting membrane (ILM) transplantation and experiencing surgical failure. **A** Persisting highly myopic macular hole (HMMH) in a patient who decided not to undergo further surgery. **B** Early HMMH recurrence in a patient in which anatomical success was visible at 1-month follow up, but a macular hole retinal detachment (MHRD) was highlighted at the 3-months follow up (central column, bottom image). Note the remainders of the ILM plug which were still partially attached to retinal boundaries (white arrow). **C** Case of late HMMH recurrence in a patient in which the ILM plug correctly sealed the hole at the 12-months follow-up. However, at month 18, the ILM plug appeared contracted toward the temporal side and a hole recurrence was identifiable

Overall, primary anatomical success was reached in 7/13 eyes (54%). Two patients (15%) with preoperative large holes (808 and 739 μm) showed persisting HMMH at 1-month follow-up but decided not to undergo further surgery. Similarly, two patients (15%) developed early recurrence at 3-months and 6-months follow-up, respectively, avoiding to undergo further operation since BCVA was stable. Finally, two patients (15%) developed late HMMH recurrence: one at the 18-months follow-up, with concomitant MHRD, and the other at the 24-months follow-up. Both of them underwent re-operation with good anatomic outcomes (Fig. 1).

**Fig. 2:**
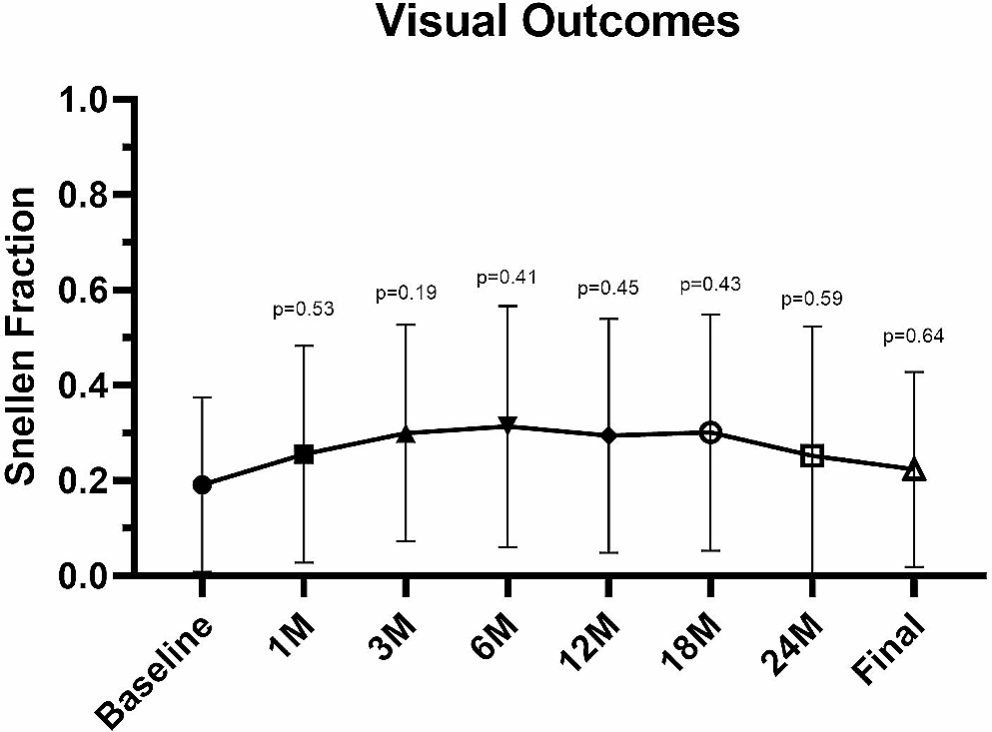
Line graph showing the evolution of best-corrected visual acuity (BCVA, Snellen fraction) through follow-ups. *P*-values indicate multiple comparisons test with Dunnet’s correction

Focusing on functional outcomes, BCVA went from 0.19 ± 0.18 (Snellen equivalent) at baseline to 0.29 ± 0.24 at 12 months, 0.25 ± 0.27 at 24 months and 0.22 ± 0.20 at final follow-up. However, no significant improvement compared to baseline was reported at any follow-up (all *p* > 0.05) (Fig. 2). Overall, at the end of the study, BCVA improved in 5/13 eyes (38%), remained stable in 3/13 eyes (23%) and worsened in 5 eyes (38%). Two eyes (ID 03 and 10) showed at least 2-lines improvement in visual acuity.

### OCT analysis and adverse events

Regarding OCT features, baseline MLD significantly correlated with the risk of refractory or early recurrent HMMH (*p* = 0.03), differently from baseline BD (*p* = 0.40). In eyes with anatomical success, foveal restoration without a visible ILM plug was reached in 3 eyes, while ILM plug was still visible in 4 eyes. However, only one eye showed continuous ELM and EZ lines, while another eye showed an irregular ELM but EZ was not visible (Supplemental file 2). The ELM/EZ complex was undetectable in any of the other eyes.

On the other hand, in patients with refractory HMMH or early HMMH recurrence (patients 07 and 09), the ILM plug was not identifiable at OCT analysis, suggesting post-operative displacement (Fig. 1A). In one patient (ID 01) who developed late HMMH recurrence, intermediate follow-up showed a visible ILM plug after 12 months. The ILM plug was no more identifiable when HMMH recurrence occurred at the 18-months follow-up (Fig. 1C). Conversely, in patient 03, no clear outer retinal restoration was visible behind the ILM plug at the 1-month follow-up. At month 3, the patient came back with a recurrent HMMH associated with MHRD, and one of the OCT scans showed how the remnants of the ILM plug were still attached to the boundaries of the detached retina (Fig. 1B).

**Fig. 3:**
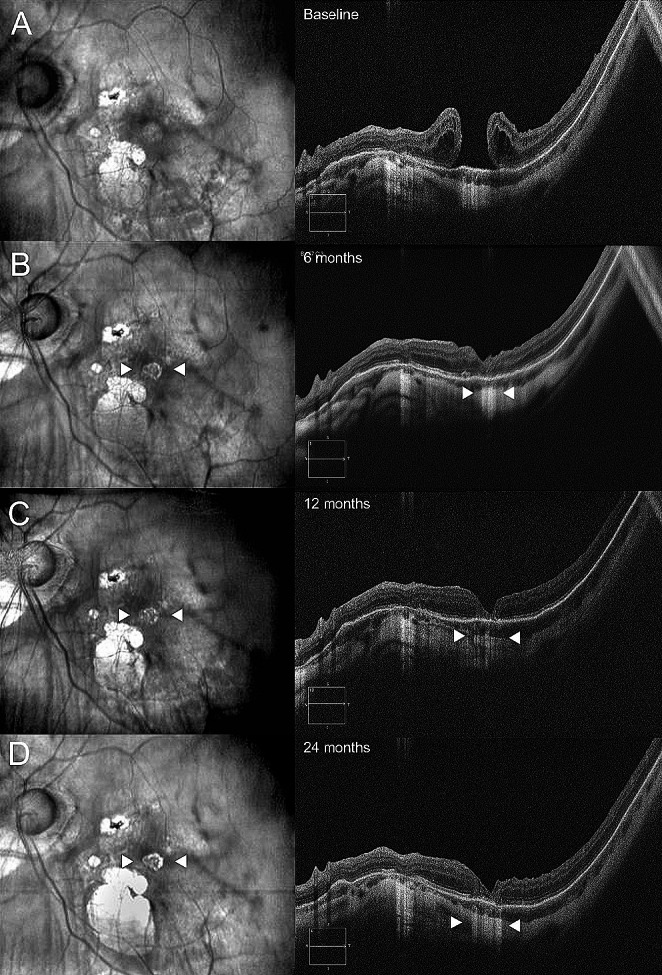
Case of sub-foveal patchy atrophy development during a 24-months follow-up. Although anatomical success, sub-foveal choroidal hypertransmission and retinal pigment epithelium (RPE) atrophy was identifiable starting from the 6-months follow-up (**B**) in infrared images (left column) and B-scans (right column). As indicated by white arrowheads, the atrophy progressively enlarged during the study period and was associated with poor visual outcomes

**Fig. 4:**
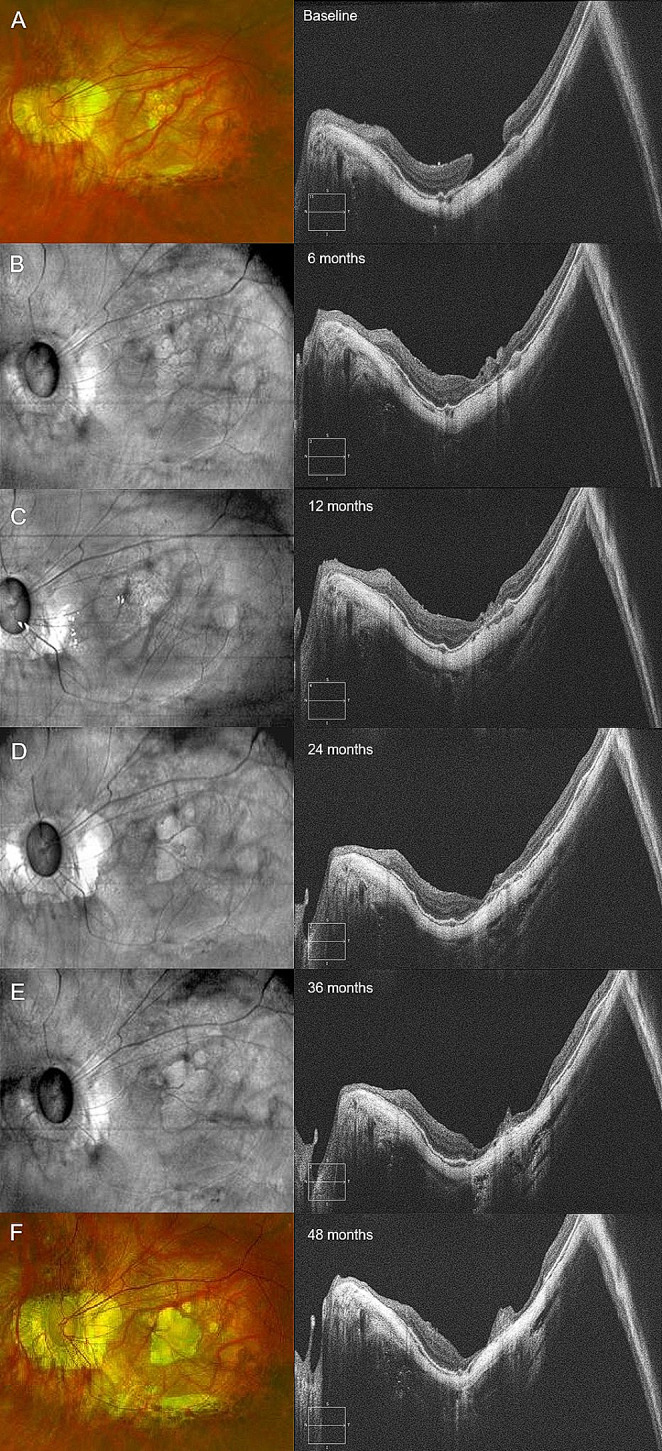
Long-term follow-up of a patient with refractory highly myopic macular hole (HMMH) showing successful closure but sub-foveal patchy atrophy development and late excessive gliosis. As visible with baseline fundus photography (**A**), infrared images at intermediate follow-ups (**B**-**E**) and final fundus photography (**F**), patchy atrophy progressively enlarged and involved the foveal area. B-scans (right column) showed the evolution of the ILM plug, which progressively shrunk till month 24 but then underwent progressive gliosis, clearly visible at 48-months follow-up (right column, bottom image)

Overall, out of the 7 eyes with favorable anatomic outcome, 5 eyes (71%) developed progressive atrophy of the subfoveal RPE, identifiable as increased choroidal hypertransmission and concomitant appearance of an atrophy patch at infrared (IR) pictures and fundus photography (Fig. 3 and 4). In all cases, RPE atrophy was identifiable between the 6-month and 12-month follow-ups and was confined to the area of the pre-existing HMMH.

**Fig. 5:**
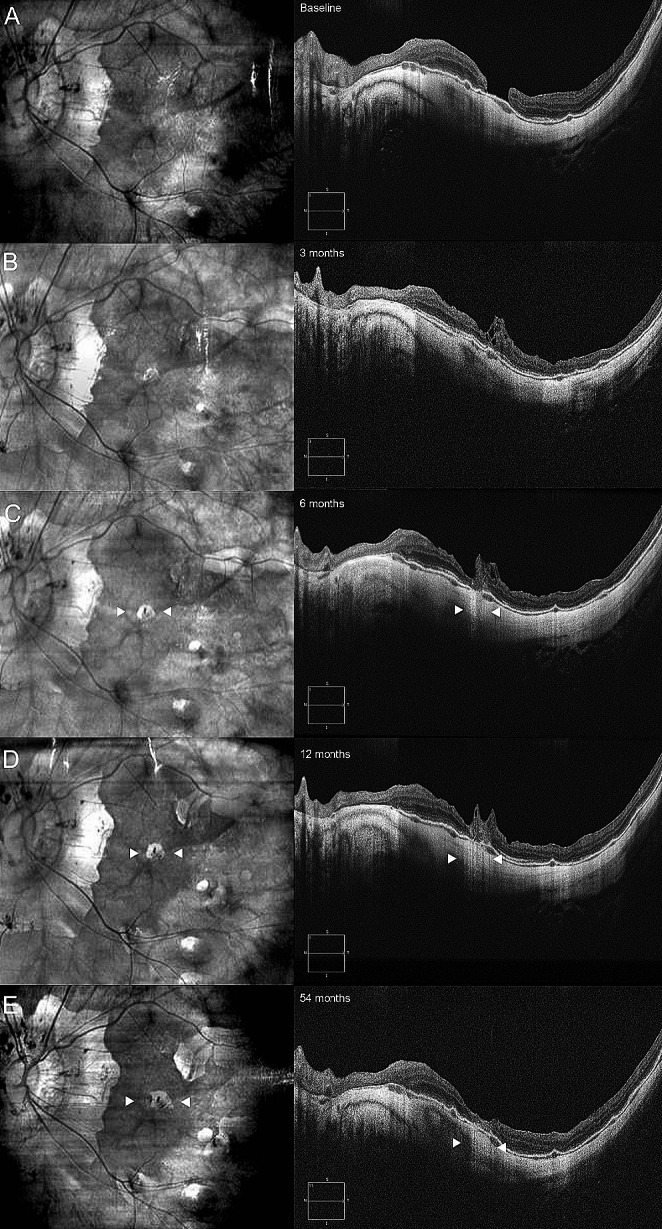
Evolution of excessive gliosis and sub-foveal patchy atrophy through a 54-months follow-up in infrared images (left column) and B-scans. Cystic degeneration of the ILM plug was visible at the 3-months follow-up (**B**, right column), and left space to increasing excessive gliosis and enlarging RPE atrophy (white arrowheads) through 6- and 12-months follow-ups (**C** and **D**). Finally, at 54-months follow-up, subfoveal atrophy had further enlarged, but excessive gliosis regressed, even if no restoration of outer retinal layers was identifiable

Excessive gliosis was reported in 3 eyes (28%). In the first case, the lesion slowly increased over time and reached stability 12 months after surgery, not being associated with RPE atrophy. In the second case, excessive gliosis appeared 36 months after surgery and was associated with subfoveal patchy atrophy (Fig. 4). Finally, in the third case (ID 02), the lesion increased in the first 12 months but concomitant subfoveal RPE atrophy developed. During the successive follow-ups, the gliosis showed progressive reduction and regression 54 months after surgery, while the RPE atrophy had progressively enlarged (Fig. 5).

**Fig. 6:**
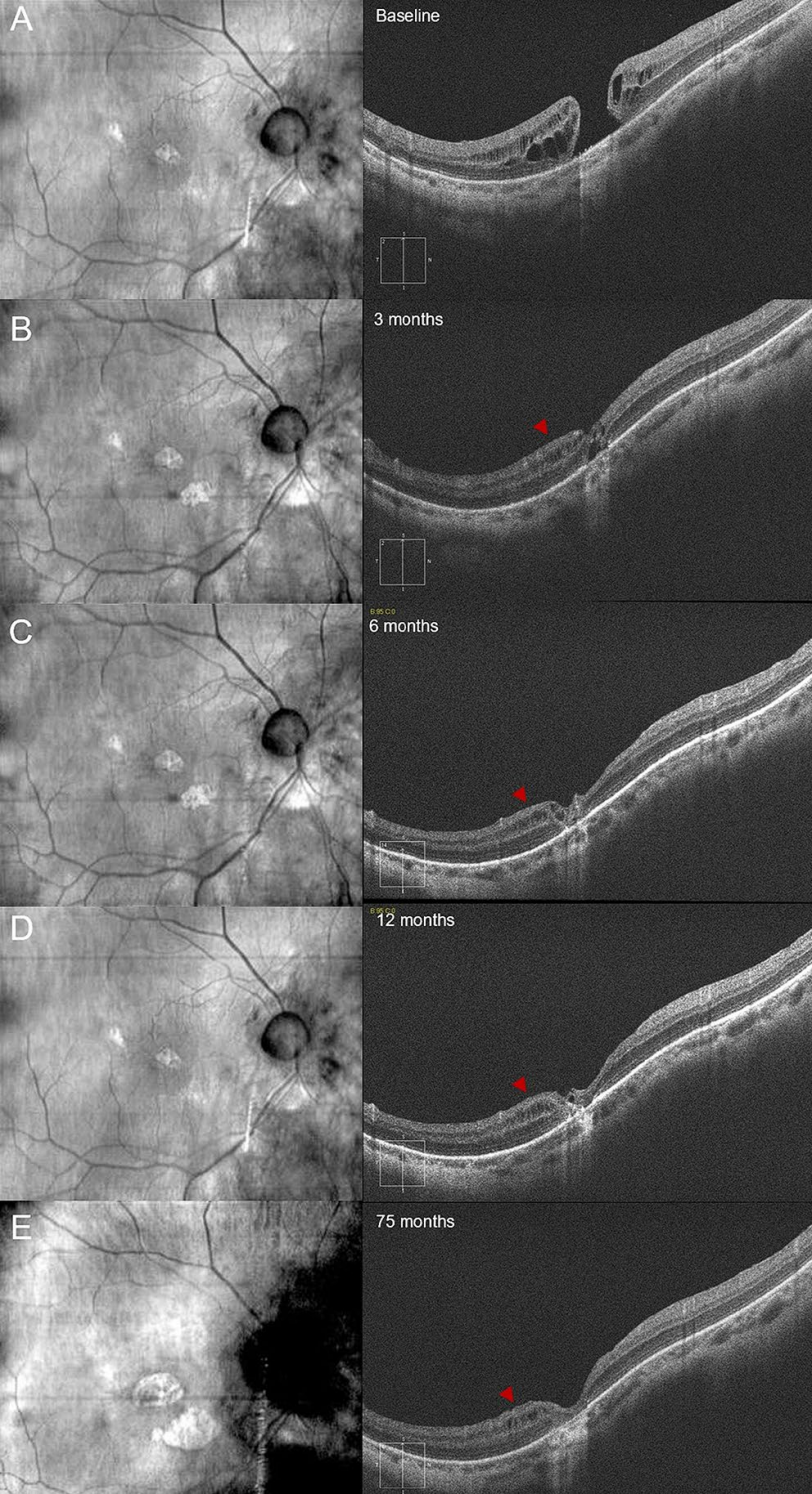
Case of persisting macular edema-like changes in the perifoveal area of a refractory highly myopic macular hole (HMMH) treated with autologous internal limiting membrane (ILM) transplantation. Microcystic changes of the inner nuclear layer (red arrowheads) appeared at the 3 months follow-up (**B**, right column) and were still visible 75 months after surgery (**E**, right column). Moreover, the patient gradually developed an enlarging sub-foveal patchy atrophy associated with poor visual outcomes

Furthermore, patient 11 developed post-operative chronic macular edema (ME)-like changes in the perifoveal area. The ME was highlighted starting from the 6-month follow-up and assumed a microcystic-like aspect, being localized in the inner nuclear layer (INL) (Fig. 6). Finally, patient 12 developed an extra-foveal myopic choroidal neovascularization (mCNV), which required 3 anti-vascular endothelial growth factor (anti-VEGF) injections.

## Discussion

Various surgical techniques based on the ‘scaffold theory’ have been proposed to treat primary and refractory MHs. These techniques include the inverted ILM flap, lens capsular flap transplantation and autologous ILM transplantation, all relying on the same healing process, which involves a membrane-like tissue that acts as a scaffold for the multiplication and movement of Müller cells. Previous research showed that the ILM flap is encircled by activated Müller cells, which also generate the same factors, and displays neurotrophic and growth factors [20]. Moreover, when Müller cells are cocultured with the ILM alone, Müller cell migration and proliferation are stimulated by ILM components of collagen IV, laminin, and fibronectin. These results corroborated the idea that adding an ILM flap considerably increases gliosis, which improves the prognosis for patients with refractory MHs [21]. 

In our research, we evaluated the role of autologous ILM flap transplantation in the treatment of recurrent HMMH. Overall, primary anatomical success was reached in 54% of cases and only 38% of eyes showed improvements in visual outcomes. In two cases, we reported anatomical failure at the first post-operative follow-up. Both patients had a preoperative large HMMH (808 and 739 μm), suggesting that this category of patients have higher risk of early displacement of the ILM plug and may require different intraocular tamponades. Rizzo et al. suggested that other tamponading agents, including “heavy” silicone oils, have been effective in closing chronic MHs [22]. However, consistent with the study of Giansanti et al., we chose a gas tamponade in our series since we looked for a high tamponading force during the initial postoperative period [23], and possible incorrect postoperative positioning may have resulted in the two cases of displacement we reported. On the other side, the use of longer acting gases (e.g. C3F8) or the use of viscoelastics substances as surgical adjuvants to hold the flap in place could have been an option to optimize post-operative anatomical outcomes [24], but was not assessed in our research.

Moreover, 4 patients developed early (in the first 6 post-operative months) or late recurrence of the HMMH, which occurred even 2 years after surgery. When compared to reports on hemmetropic refractory MHs, in which free ILM transplantation showed success rates of 91–100%, (21 − 16 di giansanti, giansanti) anatomic results in HMMH are sensibly lower. However, high myopia is a well-known risk factor for MH recurrence and our case series had a mean AXL of 31.45 mm, which may have detrimentally impacted success rates of the technique.

In patients who reached anatomical success, only one eye showed visible restoration of the ELM/EZ complex, while another eye showed the presence of an irregular ELM. None of the other eyes (71%) showed restoration of outer retinal layers, thus leading to poor visual outcomes. Giansanti et al., in their case series, observed good recovery of outer retinal layers and good visual improvement [23]. On the other side, several studies hypothesized that the closure of a recurrent MH undergoing ILM transplantation was associated with the prolonged proliferation of glial tissues in the fovea with fibrotic and depigmentation phenomena, which often hampered the restoration of outer retinal layers, consistent to the findings of our research [25, 26]. 

Moreover, starting at the 6- and 12-months follow-up, 71% of eyes developed RPE subfoveal atrophy, which was detectable in infrared OCT scans and in fundus photography, as a white roundish lesion with well-defined borders in the foveal area slowly enlarging during the study period. Previous reports in literature highlighted the occurrence of RPE atrophy after MH surgery but addressed this complication to the use of vital dyes, such as trypan blue and indocyanine green [27, 28], and, more recently, Brilliant blue [29]. Nevertheless, all these case showed a diffuse RPE atrophy involving larger areas or the entire posterior pole. In our research, the RPE atrophy had a nummular aspect, similar to patchy atrophy (PA), and progressively developed in the area of the pre-existing hole, suggesting that the ILM plug, even in cases of anatomical success, often fails to integrate with the surrounding retina and alters the homeostasis in the foveal area, eventually leading to RPE sufferance. Notably, a clinical evidence of myopic atrophic maculopathy with PA was already visible preoperatively in all of these eyes, thus suggesting a pre-existing choroidal circulation imbalance, which, combined with scleral fragility of myopic eyes and the choriocapillaris disappearance, enhances the vulnerability of the RPE, as demonstrated in previous research [30]. We hypothesize that the presence of the ILM plug, since being a scaffold of non-vascularized tissue, may be an additive element hastening the development of PA in the foveal area.

Moreover, we reported excessive gliosis in 3 eyes (43%). As previously reported for the inverted flap technique performed in HMMH, the ILM has a critical function in encouraging the hyperproliferation of Müller cells. Ye et al. recently reported a 42% rate of excessive gliosis in HMMH undergoing ILM inverted flap, showing that the risk is enhanced when AXL is more than 29.985 mm [31]. In our research, all eyes experiencing excessive gliosis had an AXL > 31 mm, consistent with the idea that AXL may be an independent factor for the development of this side effect. Previous studies claimed that highly myopic eyes have an altered ILM microstructure and Müller cells symmetry [32], and that the inverted ILM flap may induce anomalous activation of Müller cells which move along the pathologically altered ILM “scaffold” and ultimately result in excessive gliosis [33, 34]. 

In a Western blot analysis of highly myopic eyes with foveoschisis, the ILM showed increased levels of the acidic glial fibrillary protein (GFAP), a marker of reactive gliosis and stress for Müller cells [20]. Stressed Müller cells have a higher tendency to multiply quickly when damage connected to surgery takes place [35]. In refractory HMMH, already damaged Müller cells undergo further surgical stress due to intraoperative handling of the ILM transplant in the foveal area. Furthermore, we suggest that the presence of the ILM plug enhances the re-activation of Müller cells which leads to anomalous gliosis developing in the first post-operative months. As already demonstrated, excessive glial growth may have an impact on the rebuilding of the photoreceptor layer and, consequently, on visual outcomes [31, 34]. This hypothesis is consistent with our results, in which the patients developing this lesion did not show any restoration of ELM/EZ, and BCVA at the end of the follow-up period was equal to baseline.

Our study has several limitations, primarily due to the intrinsic nature of this retrospective research, which lacked a control group. Second, the sample size was relatively small and the follow-up periods were heterogeneous, even if all the patients completed at least 12 months of follow-up and in some cases we were able to study OCT changes happening 6 or 7 years after surgery. In addition, other functional outcomes, such as post-operative metamorphopsia, significantly impact quality of life and could have been investigated.

After a long term experience with HMMHs and after highlighting the shortfalls of autologous ILM flap transplantation, our group progressively abandoned this option in favor of newly proposed techniques, such as the use of hAM patches [36] and macular hydrodissection [37], which demonstrated better anatomical and functional outcomes, even if reports with long-term follow-ups are not yet available in literature. A similar process occurred with the use of macular buckling, which had been our mainstay for the management of MTM [38], but was then supplanted in our practice by less invasive techniques with lower rates of early and late complications.

In conclusion, autologous ILM transplantation with gas tamponade showed controversial results in recurrent HMMH, either in terms of anatomical and functional outcomes, with reconstitution of outer retinal layers being visible only in rare cases and a high risk of hole recurrence. Moreover, we highlighted the development of several post-operative complications, such as progressive subfoveal patchy atrophy and excessive gliosis, further limiting visual results.

## Electronic supplementary material

Below is the link to the electronic supplementary material.

Supplementary Material 1

Below is the link to the electronic supplementary material.

Supplementary Material 2

## Data Availability

The data that support the findings of this study are available from the corresponding author, MMC, upon reasonable request.
