# Supplementary figures and images for: Shortfalls of free autologous internal limiting membrane transplantation for highly myopic refractory macular holes in a long term follow-up
Source: Graefes Arch Clin Exp Ophthalmol. 2024 Jun 4;262(11):3531–41. doi: 10.1007/s00417-024-06533-7 (PMC11584440; doi:10.1007/s00417-024-06533-7)

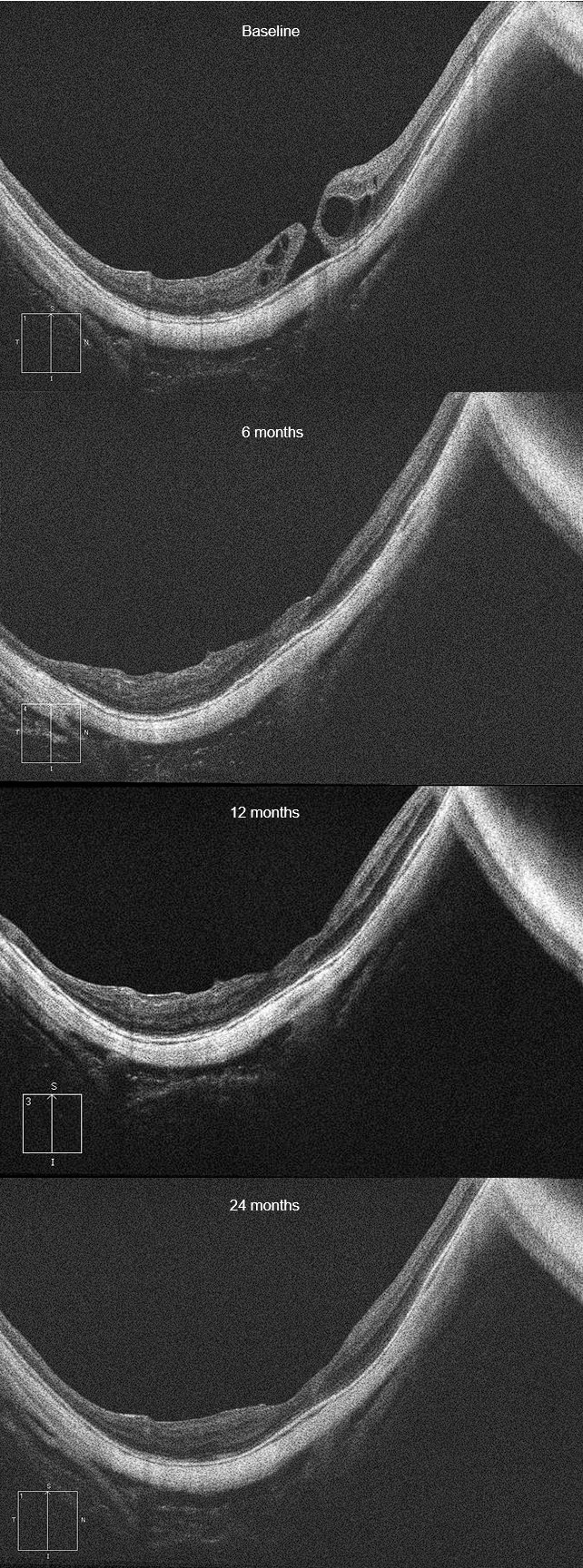

Supplement: Supplementary file 1 — Supplementary Material 1 [file 417_2024_6533_MOESM1_ESM.tiff]

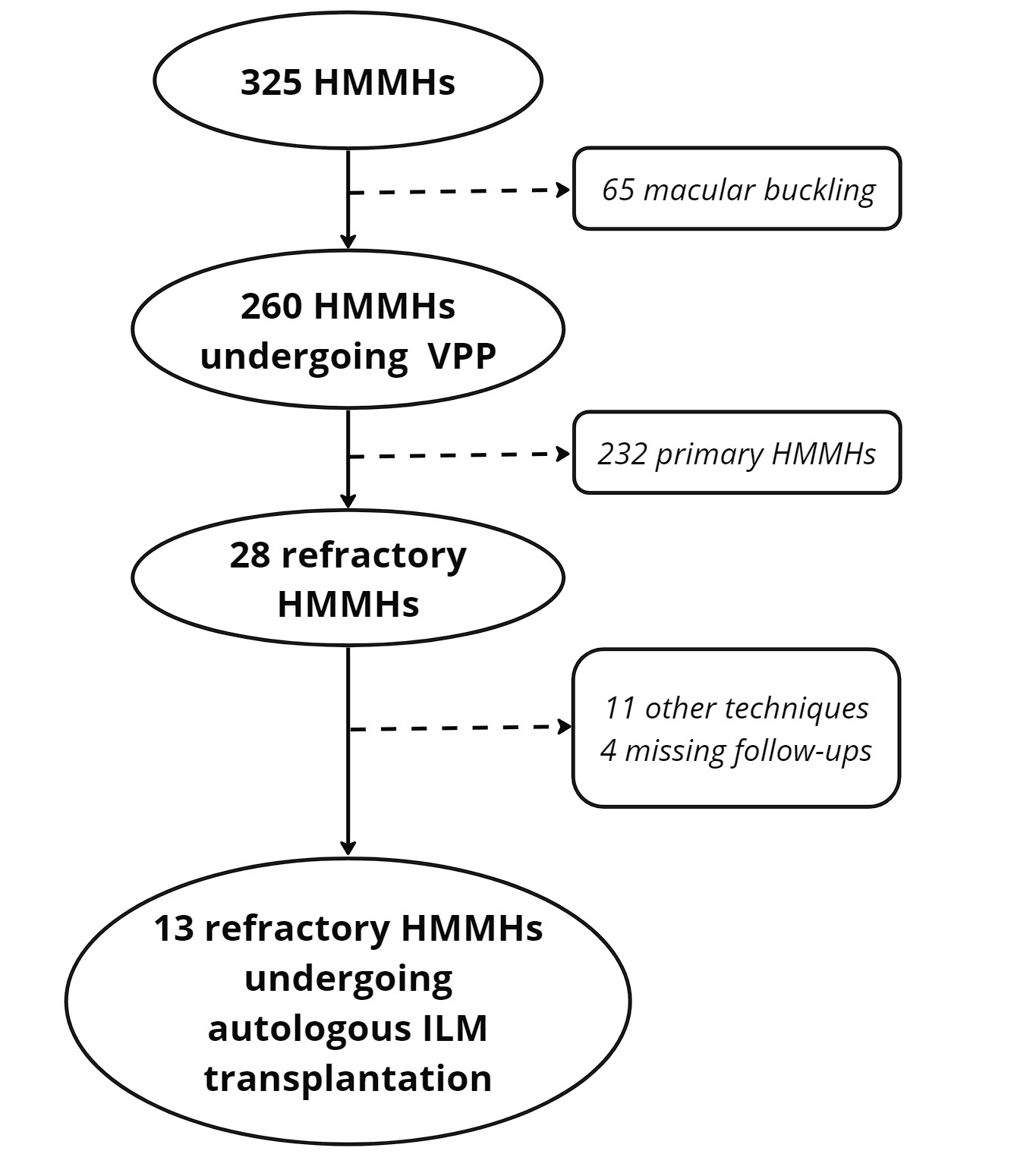

Supplement: Supplementary file 2 — Supplementary Material 2 [file 417_2024_6533_MOESM2_ESM.tiff]
